# Gold colloidal nanoparticle electrodeposition on a silicon surface in a uniform electric field

**DOI:** 10.1186/1556-276X-6-580

**Published:** 2011-11-04

**Authors:** D Buttard, F Oelher, T David

**Affiliations:** 1 CEA-Grenoble/INAC/SiNaPS-MINATEC 17 avenue des martyrs 38054 Grenoble, France; 2 Université Joseph Fourier/IUT-1 17 quai C. Bernard 38000 Grenoble, France

## Abstract

The electrodeposition of gold colloidal nanoparticles on a silicon wafer in a uniform electric field is investigated using scanning electron microscopy and homemade electrochemical cells. Dense and uniform distributions of particles are obtained with no aggregation. The evolution of surface particle density is analyzed in relation to several parameters: applied voltage, electric field, exchanged charge. Electrical, chemical, and electrohydrodynamical parameters are taken into account in describing the electromigration process.

## 1. Introduction

The emerging fields of nanoscience and nanoengineering are helping us to better understand and control the fundamental building blocks in the physics of materials [1,2]. The manipulation of nano-objects is also essential and requires expertise in several domains (mechanics, electro-chemistry, optics...) [3-5]. The traditional *top-down *approach is by far the most widespread within the micro-electronics industry, but it relies on a complex lithography technique that results in very high production costs. Alternative approaches are therefore being investigated with a view to achieving a spontaneous self-assembly of nano-components. Among these approaches, the so-called *bottom-up *method is attracting increasing attention. Based on this method, the self-organization of gold nanoparticles on a planar surface is providing new solutions for electrical or catalytic systems [6,7]. However, the deposition of particles on a substrate [8,9] must conform to several criteria such as irreversibility of the deposition process [10], stability, and high density. Deposition of gold colloidal nanoparticles can be achieved with different methods. For instance, the electrophoretic deposition method (EPD) [11,12] uses a uniform external electric field to drive the suspended particles from the solution toward the substrate surface. The advantage of the EPD method is that it requires no special surface passivation on the colloidal particles and it can be controlled conveniently by the applied field [13,14]. The deposition process, however, is complex [15] and many questions remain unanswered, despite the extensive use of EPD.

In this article, we describe the uniform electric field-assisted deposition of gold colloidal nanoparticles from an aqueous solution onto a planar silicon surface. The adsorption of nanoparticles onto silicon is described and the surface density obtained is investigated in function of the usual experimental parameters: applied voltage, electric field, and initial nanoparticle density existing in the solution.

## 2. Material and methods

**Figure 1:**
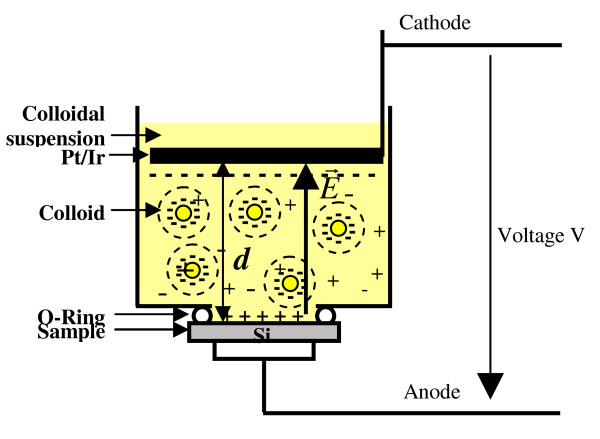
**Schematic representation of the experimental setup with negatively charged nanoparticles in the liquid solution**.

Gold colloidal nanoparticles from the British Bio Cell Company were deposited on standard *p*-type silicon wafers, <111>-oriented, with a low electrical resistivity (*ρ *< 0.01 Ωcm) to ensure a good ohmic contact in the electrochemical cell. Prior to particle deposition, the silicon wafers were deoxidized using vapor hydrofluoric acid (HF) at room temperature above a liquid HF solution with 49 vol.%. Thanks to this process, the silicon surface of the wafer is free of the native silicon oxide that usually covers a silicon surface. The colloidal solution is an aqueous-stabilized dispersion of gold nanoparticles (particle purity 99.9%) with a controlled diameter *D *in the [20-100 nm] range. The nominal value of the diameter is given by the supplier with 10% mono-dispersed. This was confirmed by electron microscopy measurements. Gold colloidal nanoparticles are stabilized by citrate ions (PH = 6.5) and exhibit a negative total charge. Gold colloidal solutions were stored at low temperature (*T *= 5°C) to prevent any unwanted aggregation. Experiments were conducted at room temperature only from fresh un-aggregated solutions. The electromigration process was performed using a homemade electrochemical set-up with a Parstat P-2273 potentiostat. Figure 1 illustrates the experimental details [both the voltage (*V*) and electrode distance (*d*) are free parameters]. Typical experiments consist in monitoring the current (*I*) versus time (*t*) at a fixed voltage (*V*), between the silicon surface (anode) and the platinum counter-electrode (cathode) in the 0.1-40 V range. Colloidal nanoparticle density on the substrate surface was evaluated afterward from scanning electron microscopy (SEM) images obtained with a FEG-SEM Zeiss ultra 55 allowing nanoscale resolution. Particle distribution statistics were performed using the ImageJ software on contrast-enhanced images. For one sample, the silicon substrate was replaced by a platinum-coated silicon substrate. The platinum material was deposited by sputtering (under a pressure *P *= 10^-7 ^mbar), resulting in a uniform 300 nm Pt layer on the silicon substrate.

## 3. Results and discussion

**Figure 2:**
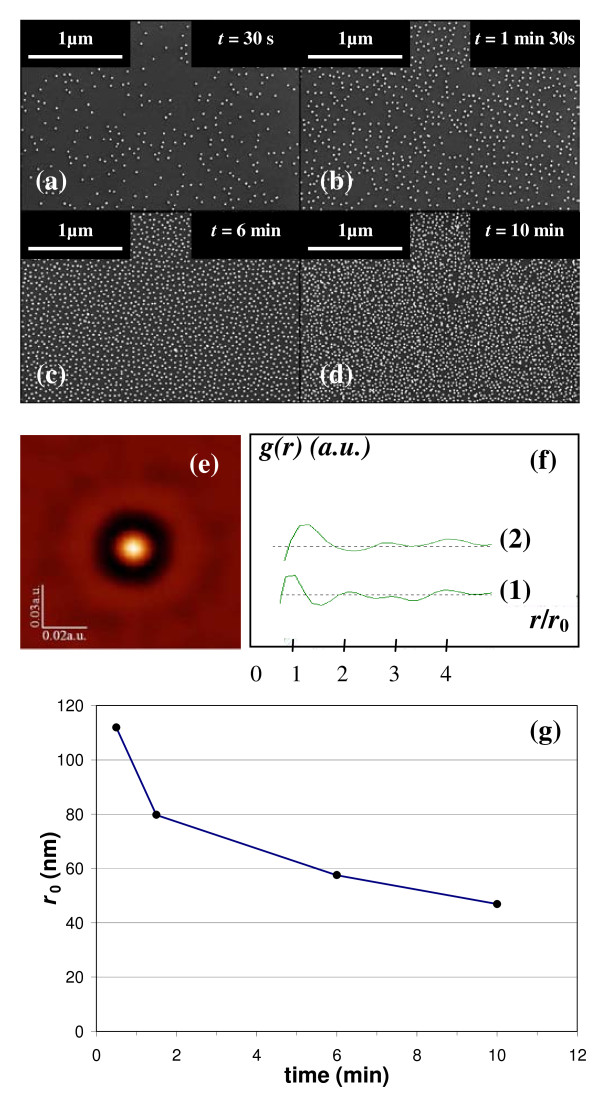
**In-plane distribution of the gold colloids**. **(a-d) **SEM plane views of a <111>-oriented silicon substrate after electromigration of gold nanoparticles with a diameter *D *= 20 nm for different deposition times *t *at a voltage *V *= 40V, **(e) **self-correlation function *g*(*r*) from **(d) **with *r*_0 _= 46.9 nm, **(f) **cross section (1) from **(e) **and (2) from **(c)**, **(g) **evolution of *r*_0 _with deposition time.

Figure 2 presents SEM images of gold colloidal nanoparticles (diameter *D *= 20 nm) electrodeposited on a silicon surface under a constant voltage *V *= 40 V for various deposition times *t*. For short deposition times (Figure 2a,b), the observed nanoparticle density is low. At longer times (Figure 2c,d), the density increases and eventually saturates. Images recorded for times longer than 10 min are similar to those of Figure 2d. After deposition had occurred, several techniques were tested to desorb the nanoparticles, such as using a reversed electric field or dipping the sample into a basic or acid bath. Following such treatment, no change in the surface density of the deposited nanoparticles was observed. This chemical and electrical stability indicates that the nanoparticles are strongly fixed to the surface, with no observable lateral mobility. As the silicon substrate corresponds to the anode, the anodic oxidation of the silicon surface occurs around the gold nanoparticles and probably leads to the partial embedding of the particles in SiO_2_. This may explain the strong adsorption of the particles at the silicon surface. Careful observation of Figure 2a-d reveals no aggregation. Particles are uniformly distributed overall the surface and are well separated from their nearest neighbors.

This is corroborated by Figure 2e, showing a typical two-dimensional self-correlation function *g*(*r*), calculated from the SEM image at *t *= 10 min. This radial distribution corresponds to the probability of finding a particle at a center-to-center distance *r *from another particle [16]. This statistical result, based on an evaluation of all particles observed on the image, confirms the uniform distribution of the nanoparticles. A profile from a *g*(*r*) cross section (Figure 2f(1)) shows several oscillations, despite the lack of periodic ordering. This cross section was normalized by *r*_0 _which corresponds to the average distance between nearest neighbors. Here, we measure *r*_0 _= 46.9 nm (abscissa of first peak of *g*(*r*)) which indicates that the 20 nm diameter nanoparticles are only separated by a surface-to-surface distance of 26.9 nm on average. We note that other peaks are clearly visible on *g*(*r*). This is evidence that, although there is no periodic distribution in the observation plane, the nanoparticles are uniformly scattered over all the substrate with a measurable nearest neighbor distance [17]. Self-correlation functions were also computed for other SEM images (Figure 2a-c). An example is shown in Figure 2f(2).

Figure 2g shows the corresponding *r*_0 _for each deposition time. As expected, *r*_0 _is long for short deposition times (low density) and saturates around 40 nm at longer deposition times. This value (at saturation) corresponds to a surface-to-surface distance *l *· 20 nm between nearest particles, which is close to the nominal particle diameter. This distance corresponds to an electrical equilibrium between charged particles. Gold colloidal nanoparticles are embedded by citrate ions leading to a negative charge at the surface of the colloids. This negative charge is balanced by the adsorption of positive ions present in the electrolyte. The electrical atmosphere around the particles is therefore very complex [18,19] and there are a lot of charge interactions between the particles. In the well-known double layer model based on the Gouy-Chapmann theory [20,21] and Stern's model [22], the particle is embedded both by a compact layer, adsorbed at the surface, and by a diffuse layer. Usually in an electrolyte, the Debye length *λ*_D _is taken as the thickness of both the compact and the diffuse layers. The Debye length is an important factor in determining the stability of gold colloid. Under appropriate conditions, particles do not coalesce. This stability is due to the repulse potential of the diffuse Debye layer when two particles come close to each other. This is greater than the attractive Van der Waals potential/force of the gold particle, which would lead to coalescence of the particles. In other words, the homogenous lateral distribution of colloids is interpreted as the repulsion between two neighbors on account of the negative shell from citrate ions.

**Figure 3:**
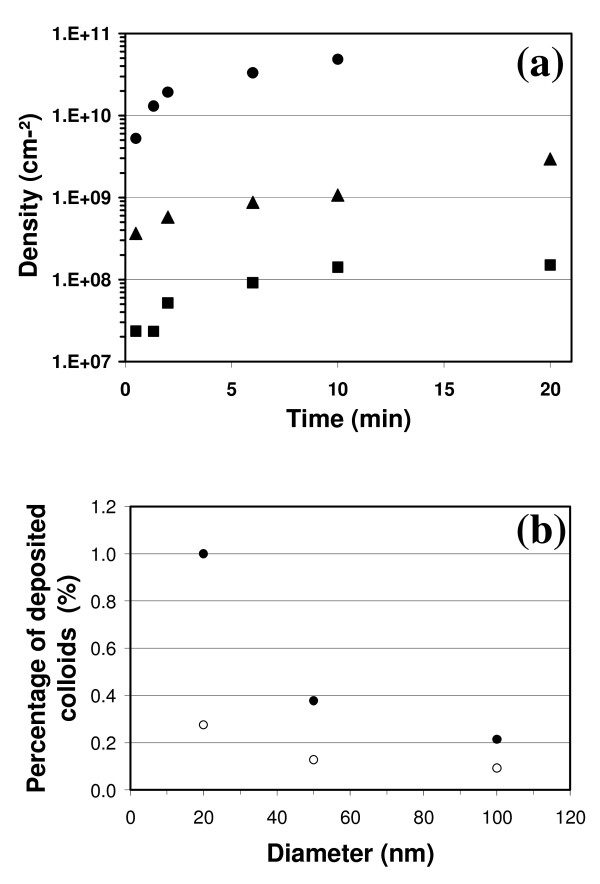
**Evolution of nanoparticle density *δ *on a <111>-oriented silicon surface under a constant voltage *V *= 40 V**. **(a) **versus a deposition time *t *for nanoparticle diameters *D *= 20 nm (full circles), *D *= 50 nm (full triangles), *D *= 100 nm (full squares), **(b) **percentage of deposited nanoparticles relative to the initial colloidal nanoparticle concentration in the liquid, after 2 min (open circles), after 10-15 min (full circles) of deposition, versus nanoparticle diameter.

To investigate the deposition process, similar experiments were performed with the colloidal suspension of particles with different diameters (*D *= 20, 50, 100 nm). Figure 3a shows the corresponding density *δ *of nanoparticles, measured from SEM images, versus deposition duration. The density evolves in a similar manner for each nanoparticle diameter: a sharp rise at the early stages of the deposition process and a saturation regime at *t *= 10-15 min. The saturation density value (*δ*_lim_) depends on nanoparticle diameter. In order to compare the efficiency levels of each deposition process, the particle density *δ *was normalized by the number of nanoparticles initially present in the entire liquid volume in the cell. As liquid volume and substrate area are always the same (*v *= 10 mL and *A *= 0.385 cm^2^), the percentage of deposited nanoparticles mainly depends on the concentration of each colloidal suspension (C_20 _= 7 × 10^11^, C_50 _= 4.5 × 10^10^, C_100 _= 5.6 × 10^9 ^mL^-1^). Figure 3b shows the percentage of deposited nanoparticles in relation to the initial number of nanoparticles in the liquid solution for short (open circle) and long (full circles) deposition times. In spite of the high nanoparticle density measured on the surface, we notice that only a few tenths of 1% of the particles are actually deposited. This value is not very surprising since Figure 2d shows that deposition saturates with a surface-to-surface distance close to particle diameter. At saturation level, no more particles are added to the surface, although the initial nanoparticle number in the liquid solution is still very high. (For example, a complete monolayer would correspond to a tiny fraction of the available gold nanoparticles in the liquid.) Therefore, the number of adsorbed particles on the surface may just be limited by geometrical distribution. Figure 3b also shows that the percentage of deposited nanoparticles increases as the diameter decreases. This phenomenon is more marked for longer deposition times, up to and including the 'saturation' regime. As the differences in particle concentration in the liquid have already been taken into account in the percentage values, the variations in deposited nanoparticle density are not solely explained by the different liquid solutions used during the experiment. So the observed dependence on diameter may be linked to the nature of the nanoparticles. As particle diameter increases, some deposition parameters such as particle mobility should change. But with this hypothesis, mobility variations would not affect the 'saturation' regime, where both slow and quick particles are able to reach the surface, which is not observed in Figure 3b. Consequently, hypotheses other than those involving mobility variations need to be considered, such as Ph or conductivity changes between the colloidal solutions, or interaction between particles. This last hypothesis is compatible with the geometrical limitation observed in Figure 2, but an accurate description of the phenomenon would require further experiments.

**Figure 4:**
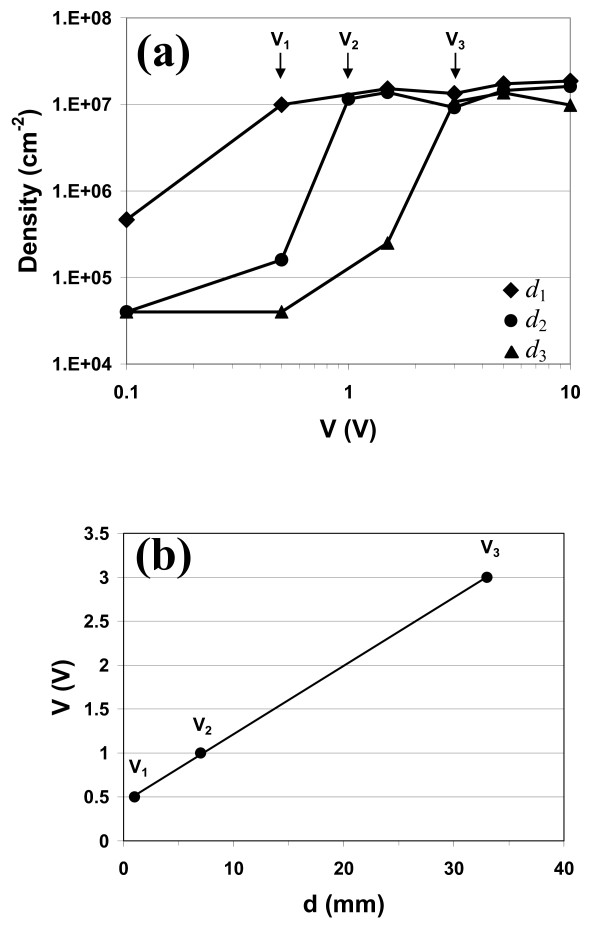
**Evolution of gold nanoparticle density (diameter D = 100 nm) versus voltage V**. **(a) **Evolution of gold nanoparticle density (diameter *D *= 100 nm) versus voltage *V *after a deposition time *t *= 1 min for three values of distance *d *between sample and electrode: *d*_1 _= 1 mm (full squares), *d*_2 _= 7 mm (Full circles), *d*_3 _= 33 mm (full triangles); **(b) **Linear evolution of the threshold voltage, *V *= 0.078 *d *+ 0.437, corresponding to a transitional electric field *E *= 78 V/m.

**Figure 5:**
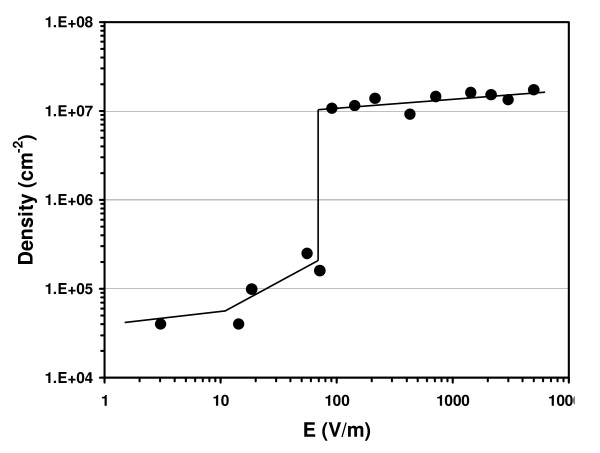
**Gold nanoparticle density (diameter *D *= 100 nm) on the silicon surface versus the uniform electric field *E *= *V*/*d***. A sharp increase in density is observed for *E*_trans _≈ 80 V/m.

As long deposition time results did not affect the deposition process itself, we investigated nanoparticle deposition with small voltages and short deposition times. Figure 4 shows measurements of particle density (diameter *D *= 100 nm) versus voltage for three different electrode positions (*d*_1 _= 1, *d*_2 _= 7, *d*_3 _= 33 mm) after 1-min deposition time. For low voltages (*V_i _*< 1 V), density is very low (*δ *≈ 4.5 × 10^4 ^cm^-2^) and increases as the voltage increases. For high voltages (*V *> 1 V), density is clearly higher with a value of *δ *≈ 10^7 ^cm^-2^. Each curve shows a sharp increase in density (two orders of magnitude) at a specific voltage (*V*_1_, *V*_2, _*V*_3_). The dependence of this threshold voltage on the electrode distance (*d*) is plotted in Figure 4b and exhibits a linear evolution: *V *= 0.078*d *+ 0.437. The offset *V*_0 _= 0.437 V is linked to a residual voltage in the electrical circuit at *d *= 0. The slope of this curve corresponds to a transition electric field (*E*_trans _= 77.8 V/m) which exists between the two electrodes. Based on this observation, Figure 5 plots nanoparticle density versus the electric field *E *= *V*/*d*. As expected, the density is low (*δ *≈ 4.5 × 10^4 ^cm^-2^) for low electric field values (*E *< 10 V/m) and more than two orders of magnitude higher (*δ ≈ *1 × 10^7 ^cm^-2^) for high *E *values (*E *> 100 V/m). All the previous data collected from different experiments clearly indicate that the sharp increase in density is controlled by a minimum electric field, *E*_trans _≈ 80 V/m. Additional experiments were performed where the deoxidised Si<111> substrate was replaced by an oxidised substrate. In this configuration, no nanoparticle deposition was observed even at high electric field values (*E *> 800 V/m). Similarly, a metallic conductive Pt-coated Si substrate was used as the anode but it still did not show any sign of nanoparticle deposition. These experiments indicate that the electric field alone is not sufficient for deposition of nanoparticles to take place on the surface.

**Figure 6:**
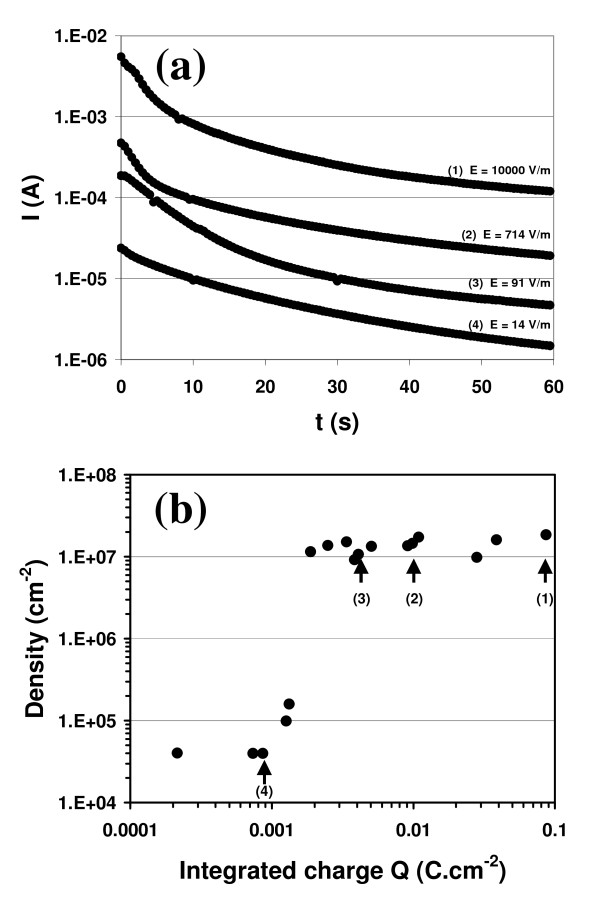
**Electrodeposition of gold nanoparticles (D = 100 nm)**. **(a) **current monitoring versus deposition time for different electric fields, **(b) **nanoparticle density versus the integrated charge *Q *exchanged between the electrolyte and the silicon surface. Points (1)-(4) match the corresponding curves of panel **(a)**. A sharp increase in density is observed for *Q *≈ 1 mC/cm^2^.

Based on this dependence on the electrode, the change in current in relation to time was investigated during the deposition time on deoxidised Si<111>*p*-type substrates. Figure 6a shows the corresponding *I*(*t*) curves with a regular decrease for all electric fields. The exchange of charges at the electrolyte/silicon interface can be characterized by the integrated total charge *Q *per surface unit exchanged during electro-deposition:

(1) Q= ∫ j(t)dt

where *j *is the current density and *dt *is the experimental time increment between two experimental points (0.5 s). Figure 6b shows the nanoparticle density versus the integrated charge *Q *(normalized by the sample surface). We observe a clear charge threshold above which density increases by two orders of magnitude. For low *Q *values (*Q *< 1 mC/cm^2^), the density is low (*δ *≈ 4 × 10^4 ^cm^-2^), whereas for high *Q *values (*Q *> 2 mC/cm^2^) the density is high (*δ *≈ 1 × 10^7 ^cm^-2^). Between these two regimes a clear transition charge threshold is observed at *Q *≈ 1.5 mC/cm^2^. We explain this behavior by the anodic oxidation of the silicon substrate, whereas the platinum is chemically inert at these voltages.

In the light of our results, we therefore propose a basic model to explain the electromigration of gold colloidal nanoparticles. In the absence of an electric field, nanoparticles are subject to colloidal forces, without any gravitational force, and the small particles are suspended in the solution. Particle transport is governed solely by Brownian's motion with random displacement. Under the influence of an electric field, particle motion occurs in a direction determined by electrophoretic parameters: electrostatic charge and solvent viscosity. The electrostatic force *F*_E _= *q*_s_*E *[23], with *q*_s _the surface charge, can only drive the negatively charged nanoparticles toward the positive electrode if a sufficient electric field overcomes the repulsive particle-particle interactions. Although our measurements (*E*_trans _≈ 0.8 V/cm) are in good agreement with the literature (*E*_trans _≈ 1.3 V/cm) [11,24,25], *F*_E _is not sufficient to explain nanoparticle transport under a uniform electric field since no deposition occurs on a Pt-coated or oxidized silicon surface. Previous investigations [14] showed that electroosmotic [26] and electrohydrodynamic [27] transport processes can direct the motion of small particles. In accordance with the literature [28], we propose here that silicon anodic oxidation takes place on the silicon anode for *V *> 1 V. The basic process of anodic oxidation at the silicon/electrolyte interface in an aqueous solution under a voltage *V *takes place as follows:

(2) $${\text{H}}_{\text{2}}\text{0}\to \text{2}{\text{H}}^{+}+{\text{O}}^{\text{2}-}$$

(3) $$\text{Si}\to \text{S}{\text{i}}^{\text{4}+}+\text{4}{\text{e}}^{-}$$

which leads to the creation of silicon oxide:

(4) $$\text{S}{\text{i}}^{\text{4}+}+\text{2}{\text{O}}^{\text{2}-}\to \text{Si}{\text{O}}_{\text{2}}$$

At the same time, hydrogen is formed at the cathode:

(5) $$\text{2}{\text{H}}^{+}+\text{2}{\text{e}}^{-}\to {\text{H}}_{\text{2}}$$

Under these conditions, a hydrodynamical flow of charged ionic species is set up in the direction of the positive electrode and this helps drive the nanoparticles toward the silicon surface. Consequently, both electrical (*E *> 80 V/m) and electrochemical parameters (*Q *> 1 mC/cm^2^) are essential to the electromigration of gold colloidal nanoparticles onto the silicon surface.

## 4. Conclusions

In this study, we have investigated the electrodeposition of gold colloidal nanoparticles on *p*-type-doped Si surfaces. Uniform distribution was obtained and adsorption was irreversible. The density of a gold nanoparticle assembly was investigated and analyzed in relation to several parameters such as voltage, the electric field, and the charge exchanged. Deposition was found to be associated with a minimum electric field (*E*_trans _≈ 80 V/m) combined with an electrochemical process (*Q *> 1 mC/cm^2^) that oxidises the surface of the Si anode.

## Abbreviations

EPD: electrophoretic deposition; HF: hydrofluoric acid; SEM: scanning electron microscopy.

## Competing interests

The authors declare that they have no competing interests.

## Authors' contributions

DB designed the experiments, performed data analysis, drafted the manuscript and supervised the whole study. FO and TD performed the experiments and participate in the manuscript. All authors read and approved the final manuscript.
